# Apparent Diffusion coefficient (ADC), T1 and T2 quantitative indexes of the myocardium in athletes before, during and after extreme mountain ultra-marathon: correlation with myocardial damages and inflammation biomarkers

**DOI:** 10.1186/1532-429X-18-S1-O41

**Published:** 2016-01-27

**Authors:** Magalie Viallon, Kevin Moulin, Caroline Le Goff, Juliette Didier, Ruud B van Heeswijk, Matthias Stuber, Charles de Bourguignon, Laurent Gergelé, Grégoire P Millet, Olivier Beuf, Pierre Croisille

**Affiliations:** 1 CREATIS UMR5220 INSERM 1044, University of Lyon, Lyon, France; 2 Radiology Department, CHU de Saint Etienne, Saint Etienne, France; 3 Siemens Healthcare, Paris, France; 4 Clinical chemistry, University hospital of Liège, Liège, Belgium; 5 CIBM/CHUV Radiology Department, University of Lausanne, Lausanne, Switzerland; 6 Anesthesiology Department, CHU de Saint Etienne, Saint Etienne, France; 7 Institute of Sports Sciences, University of Lausanne, Lausanne, Switzerland

## Background

Previous MRI and US studies have shown the existence of functional and biochemical alterations in the myocardium after prolonged endurance exercise, demonstrating transient diastolic dysfunction [1]. Simultaneous transient increases of cTnT and NT-proBNP biomarkers have been reported [2, 3] without focal necrosis identified by delayed enhancement imaging, probably due to a cytosolic's dropping of biomarkers rather than destruction of myocytes. Inflammation, microstructural & functional modifications caused by extreme loading conditions, have never been explored using quantitative MRI.

## Methods

We prospectively studied 50 runners enrolled on the 2014 « Tor des Géants » edition (the most extreme mountain ultra-marathon (336 km length, 24000 m cumulative elevation), without clinical evidence of personal history of cardiac or pulmonary disease. Subjects were studied before, at arrival, and after 3 days recovery. Imaging protocol included global and regional LV function analysis and quantitative MRI : T1, T2 and ADC values were obtained using respectively a MOLLI sequence, a radial multi-echo sequence, a Stejskal-Tanner diffusion sequence [4]. T1, T2 and ADC values at 1.5T were compared with plasma levels of inflammation, myocardial stress and/or damage biomarkers including hs-TnT, NT-proBNP, Gal-3 (a carbohydrate binding lectin produced by macrophages, upregulated in hypertrophied heart, emerging as a mediator for fibrosis development and remodeling) and ST2 (a family member of IL-1 receptors known for its role in immunological processes, having a potential role in cardiac pathogenesis).

## Results

**Figure 1:**
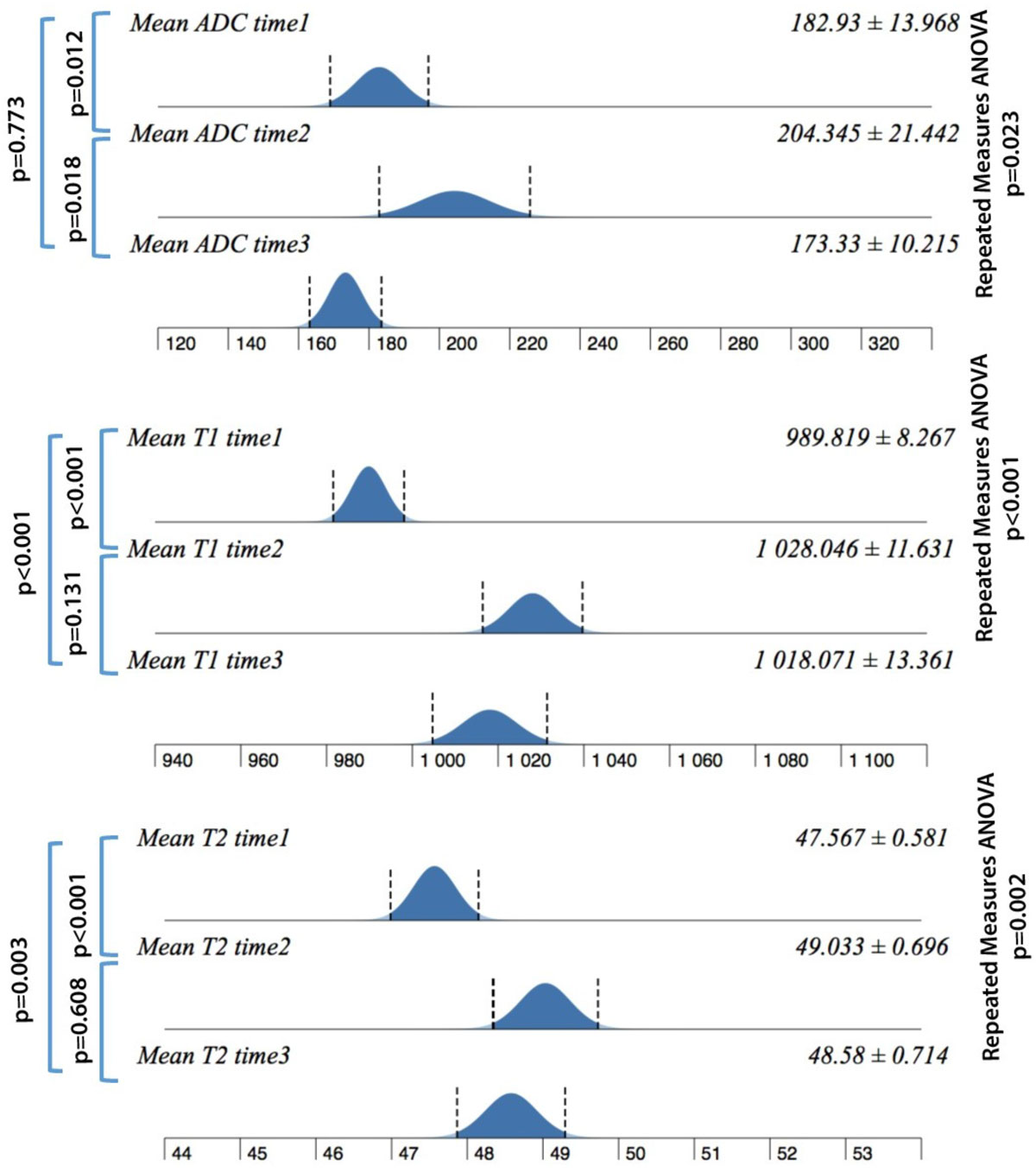
**T1, T2 and ADC sample distributions before, immediately and after 3-days recovery**.

**Figure 2:**
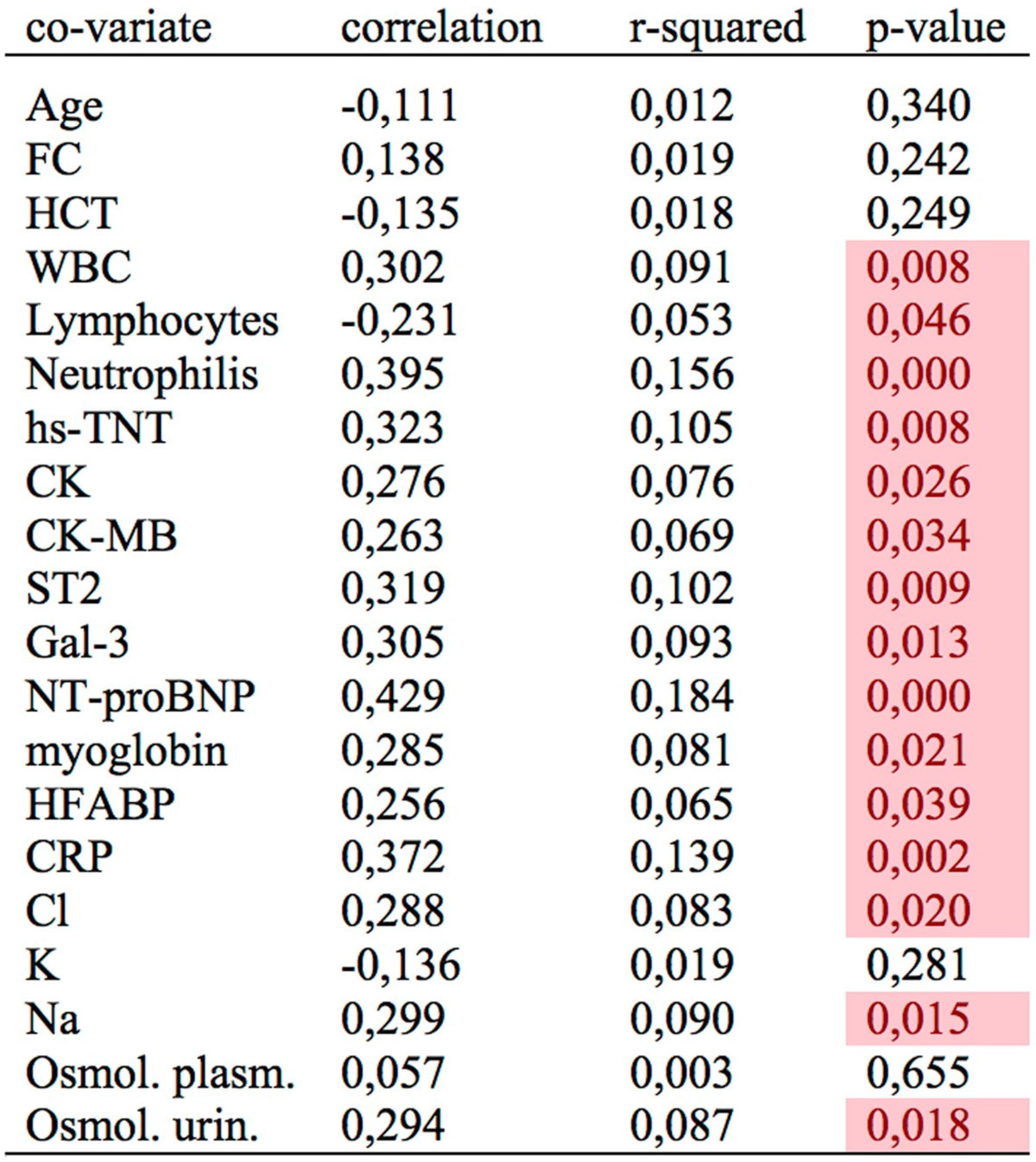
**Univariate analysis**. ADC values vs blood biomarkers. Significant correlations (p-values <0.05) are highlighted with colored overlay. FC = cardiac frequency, HCT = hematocrit, WBC = white blood cell, HFAPB= heart failure acid binding protein, CRP = c-reactive protein.

27 finishers (54%) completed the longitudinal study.T2, T1 and ADC values significantly increased immediately after the race. ADC quickly normalized after recovery while T1, T2 markers remained higher than baseline (Figure 1). Significant correlations were found between myocardial MR biomarkers and blood (Gal3,ST2,NT-proBNP), plasmatic (CRP, CKs, hs-TNT) and cellular (WBC, lymphocytes, neutrophilis) ones (Figure 2).

## Conclusions

It is the first study investigating the role of quantitative MR diffusion to explore human acute stress in humans together. ADC, T1 and T2 were all able to identify changes in subjects and related to several plasmatic biomarkers and therefore appear as valuable MR biomarkers of myocardial inflammation at least for this specific type of acute stress. Prior to a deeper understanding of the impact of ultra-endurance, this study hightlights an added value of ADC, that differ from T1 and T2 markers, to scrutinize acute stress phenomena in the myocardium. ADC represents a novel information, revealing more about water redistribution leading to ultraexercise-induced reversible myocardial inflammation. Overal it illustrates the usefulness and complementary nature of ADC as an emerging cardiac biomarker, foreseen to be deployed at short-term in the evaluation of innovative therapeutic strategies targeting inflammation.
